# The Dual Role of *Bacillus* sp. KKU-RE-018 Isolated from Medicinal Plants in Controlling Anthracnose Disease and Enhancing the Growth of Chili Plants

**DOI:** 10.3390/plants14193010

**Published:** 2025-09-29

**Authors:** Thanawan Gateta, Wasan Seemakram, Thanapat Suebrasri, Saranya Chantawong, Chaiya Klinsukon, Jindarat Ekprasert, Sophon Boonlue

**Affiliations:** 1 Department of Microbiology, Faculty of Science, Khon Kaen University, Khon Kaen 40002, Thailand; thanawan.gateta@kkumail.com (T.G.); jindaek@kku.ac.th (J.E.); 2 Department of Microbiology and Parasitology, Faculty of Medical Science, Naresuan University, Phitsanulok 65000, Thailand; wasans@nu.ac.th; 3 Division of Basic and Preclinical Science, Institute of Science and General Education, Nakhon Ratchasima College, Nakhon Ratchasima 30000, Thailand; s.thanapat@nmc.ac.th; 4 School of Preclinical Sciences, Institute of Science, Suranaree University of Technology, Nakhon Ratchasima 30000, Thailand; saranya.ch@sut.ac.th; 5 Department of Innovative Agriculture, Faculty of Agriculture, Khon Kaen University, Khon Kaen 40002, Thailand; chaikl@kku.ac.th

**Keywords:** endophytic bacteria, *Bacillus* spp., plant disease, biocontrol, plant growth promoter

## Abstract

Chili (*Capsicum annuum* L.) is a herbaceous vegetable grown and consumed worldwide. In Thailand, chili plants are severely hampered by anthracnose disease, leading to severe yield losses. This study aimed to investigate endophytic bacteria (EPB) for their potential as a biocontrol agent and plant growth promoter (PGP). Among a total of 108 isolates, strain KKU-RE-018 was identified by partial 16S rRNA gene sequencing as belonging to the genus *Bacillus*. This isolate exhibited strong antifungal activity against *Colletotrichum capsici*; its activity occurred through the production of hydrolytic enzymes, including chitinase and β-1,3-glucanase, and exhibited PGP properties. This endophytic bacterium significantly reduced anthracnose severity compared with the control, achieving a disease reduction index (DRI) of over 60%. Moreover, chili plants treated with the bacterium showed higher plant growth parameters under greenhouse conditions. The levels of phenolic compounds and salicylic acid in plants treated with *Bacillus* sp. KKU-RE-018 could activate systemic acquired resistance (SAR). Taken together, these findings demonstrate that *Bacillus* sp. KKU-RE-018 plays a multifaceted role, capable of suppressing anthracnose and simultaneously promoting chili growth.

## 1. Introduction

Chili (*Capsicum annuum* L.), an annual herbaceous vegetable [1], is a rich source of protein, vitamins A, C, and E, fiber, capsidiol, and capsaicin. It plays a significant role in domestic consumption and international trade, particularly in tropical and subtropical regions such as Southeast Asia [2,3]. In Thailand, chili cultivation contributes substantially to the country’s agricultural economy [4]. However, its cultivation is severely hampered by anthracnose disease, caused predominantly by *Colletotrichum* spp., one of the most destructive diseases affecting chili at the postharvest stages. This disease leads to fruit rot, considerable yield loss, and reduced marketability, posing a serious threat to sustainable chili production [5].

Integrated disease management (IDM), including cultural, biological, and chemical control methods, has been employed to control anthracnose. While chemical control has been effective, its prolonged and excessive use has raised concerns over environmental contamination, human health risks, and the emergence of fungicide-resistant pathogen strains [6]. In recent years, biological control has emerged as a promising alternative that aligns with sustainable agriculture. These methods are increasingly favored for plant disease management because they are economically viable, environmentally sustainable, and operationally practical.

Endophytic bacteria (EPB) are symbiotic bacteria that inhabit plant roots, leaves, and stems, benefiting the plant through growth promotion and suppressing plant pathogens [7]. *Bacillus* spp., a well-known PGPR, has demonstrated broad-spectrum antifungal activity through a variety of mechanisms, including the production of cell wall-degrading enzymes (CWDEs) such as chitinase [8,9] and β-1,3-glucanase [10,11]. In addition to their direct antagonistic activity, *Bacillus* strains are also capable of inducing systemic acquired resistance (SAR) in host plants, thereby enhancing defense responses [12,13]. Moreover, several species of *Bacillus* spp. can increase plant growth through their properties, including the production of phytohormones, such as indole-3-acetic acid (IAA) and gibberellic acid (GA_3_) [14,15], and they also play a role in the production of ammonia and siderophores and the solubilization of phosphate [16,17,18]. These multifaceted mechanisms not only enhance plant vigor but also contribute to reduced disease severity.

Although numerous studies have reported the potential of *Bacillus* strains as effective biocontrol and plant growth-promoting agents, comprehensive evaluations of isolates that simultaneously exhibit strong enzymatic activity, promote plant growth, and induce host defense responses are still limited. [19,20]. Therefore, this work aimed to investigate the efficacy of EPB in their multifaceted role against anthracnose caused by *C. capsici* and in promoting plant growth in chili plants under greenhouse conditions. This study provides insights into the enzymatic functions and defense-inducing potential of EPB, supporting their potential application as sustainable biocontrol and biofertilizer agents in chili cultivation.

## 2. Results

### 2.1. Isolation of EPB

A total of 108 EPB were isolated from the leaves, shoots, and root tissues of the medicinal plants, collected from the dry dipterocarp forest in Kalasin, Roi-et, and Mukdahan Provinces in Thailand. All bacterial endophytes were tested for their antifungal activity against *C. capsici* using a dual culture test.

### 2.2. Screening EPB Strains for Antagonistic Activity In Vitro

**Figure 1:**
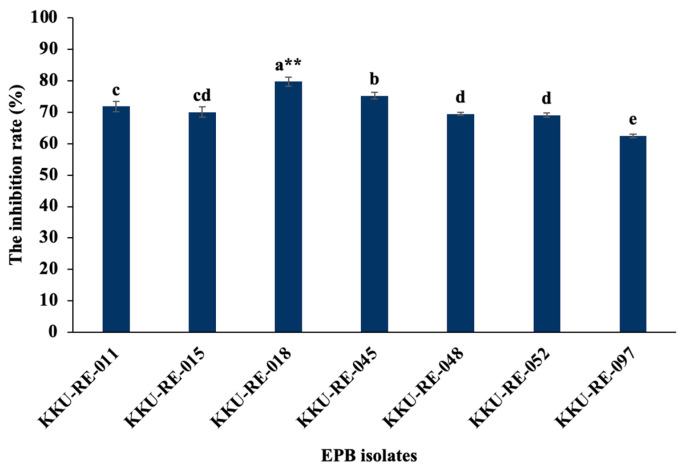
Inhibition rates of selected EPB isolates against the fungal pathogen *C. capsici* in a dual culture assay. Different letters on the bars indicate significant differences in the mean values. **, significantly different (*p* ≤ 0.01 using the LSD test).

The antifungal activity of seven strains of the isolated EPB was assessed in a dual culture plate assay. The seven strains (KKU-RE-011, KKU-RE-015, KKU-RE-018, KKU-RE-045, KKU-RE-048, KKU-RE-052, and KKU-RE-097) exhibited varying levels of inhibition of *C. capsici* growth relative to the control. The percentage of inhibition of the different strains was 71.88%, 70.00%, 79.78%, 75.25%, 69.45%, 69.11%, and 62.5%, respectively, after 7 days of incubation as compared to the control (Figure 1). Strain RE-KKU-18 exhibited the greatest level of inhibitory activity against the mycelial growth of *C. capsici*, and its inhibition level increased with the incubation time.

### 2.3. In Vitro Screening of Endophytic Fungi for Enzyme Activity and PGP Properties

**Figure 2:**
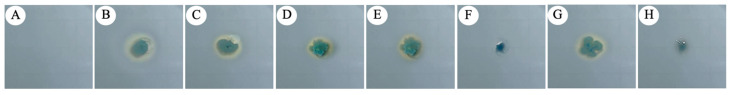
Siderophore production on CAS agar plates of EPB isolates: (**A**) control (noninoculated); (**B**) KKU-RE-011; (**C**) KKU-RE-015; (**D**) KKU-RE-18; (**E**) KKU-RE-045; (**F**) KKU-RE-048; (**G**) KKU-RE-052; and (**H**) KKU-RE-097.

All EPB strains were evaluated for their enzyme activity and PGP properties, including β-1,3-glucanase, chitinase, phosphate solubilization activity, IAA production, ammonia production, siderophore production, and HCN production, as shown in Table 1 and Table 2. The highest activity of chitinase and β-1,3-glucanase was observed with KKU-RE-018 (1.98 ± 0.05 and 1.78 ± 0.07 U·mL^−1^, respectively). In terms of their PGP properties, the highest solubilized phosphate was observed for strain KKU-RE-015 at 1067.13 µg·mL^−1^, followed by KKU-RE-18 at 918.52 µg·mL^−1^ and KKU-RE-52 at 861.11 µg·mL^−1^. In addition, the highest IAA production of NB supplemented with 0.2% L-tryptophan was found in strain KKU-RE-097 at a value of 19.35 µg·mL^−1^, followed by KKU-RE-048 at 10.69 µg·mL^−1^ and KKU-RE-045 at 8.52 µg·mL^−1^, while NB without 0.2% L-tryptophan was found in strain KKU-RE-018 at 15.10 µg·mL^−1^, followed by 13.13 µg·mL^−1^ and 11.81 µg·mL^−1^ in strains KKU-RE-97 and KKU-RE-015, respectively. The highest amount of ammonia production was observed at 42.15 µg·mL^−1^, followed by 38.70 and 37.51 µg·mL^−1^ in strains KKU-RE-045, KKU-RE-048, and KKU-RE-011, respectively. According to the ability of EPB to produce siderophores and HCN, the results revealed that the EPB isolates KKU-RE-011, KKU-RE-015, KKU-RE-018, KKU-RE-045, and KKU-RE-052 exhibited siderophore production on CAS agar media from blue to orange (Figure 2). However, HCN production was not observed in any of the EPB strains.

### 2.4. Molecular Identification of Bacterial Endophytes

**Figure 3:**
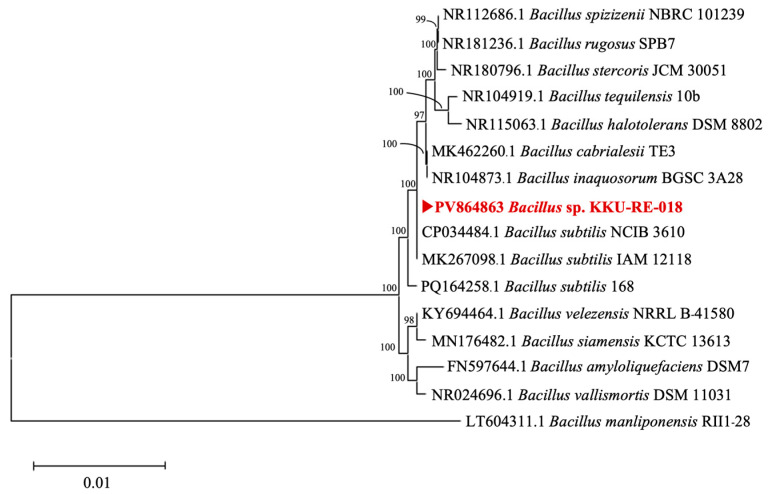
Phylogenetic tree of *Bacillus* sp. KKU-RE-018 using MEGA 11 software with the maximum likelihood (ML) method. The bacterial species identified in the present study is indicated in bold red font.

Among the tested isolates, strain KKU-RE-018 exhibited the strongest antagonistic activity against *C. capsici*, the highest levels of hydrolytic enzyme production, and notable PGP traits. Therefore, this strain was selected for molecular identification based on the partial sequence of the 16S rRNA gene. The result revealed that strain KKU-RE-018 showed 100% similarity to *Bacillus* spp. (GenBank accession no. PV864863). The phylogenetic tree further confirmed its close clustering with the genus *Bacillus* (Figure 3).

### 2.5. Scanning Electron Microscope Observation of C. capsici Changes in Mycelia Morphology

**Figure 4:**
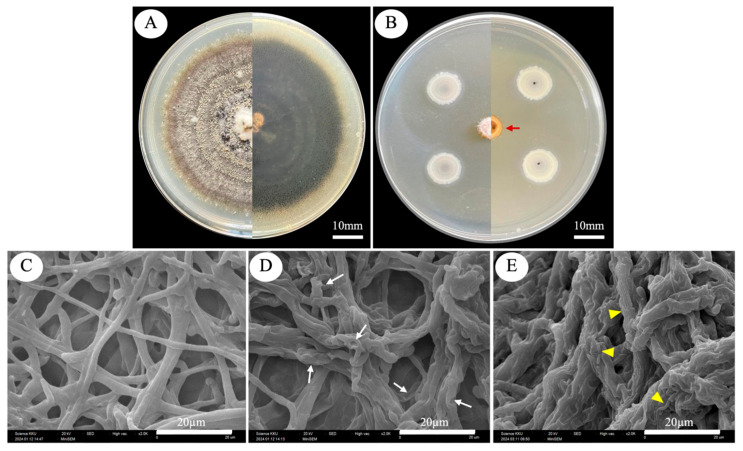
Antifungal activity tested using a dual culture assay (top) and SEM micrographs of *C. capsici* hyphae (bottom): (**A**) control pathogen fungal *C. capsici*; (**B**) inhibitory effect of *Bacillus* sp. KKU-RE-018 against *C. capsici* after 7 days of inoculation, showing an inhibition zone (red arrow); (**C**) intact hyphae of fungus on control plate; (**D**) distortion and destruction in the hyphae of *C. capsici* (as indicated by the white arrow); and (**E**) presence of bacterial spores attached to fungal hyphae, with severe hyphal collapse (yellow arrowheads).

*Bacillus* sp. KKU-RE-018 displayed good inhibitory potential against *C. capsici*, as shown by the morphological changes in fungal mycelia (Figure 4). The results showed that the hyphae of *C. capsici* co-cultured with *Bacillus* sp. KKU-RE-018 exhibited perforations, loss of turgidity, and shrinkage. Collapsed and dead hyphae were also observed (Figure 4D, white arrows). Notably, in Figure 4E, bacterial spores were observed attached to the fungal hyphae (yellow arrowheads), coinciding with severe hyphal collapse. No morphological changes in the mycelia were observed in the control set.

### 2.6. Biocontrol Potential of Bacillus sp. KKU-RE-018 on the Anthracnose Severity of Chili Fruit

**Figure 5:**
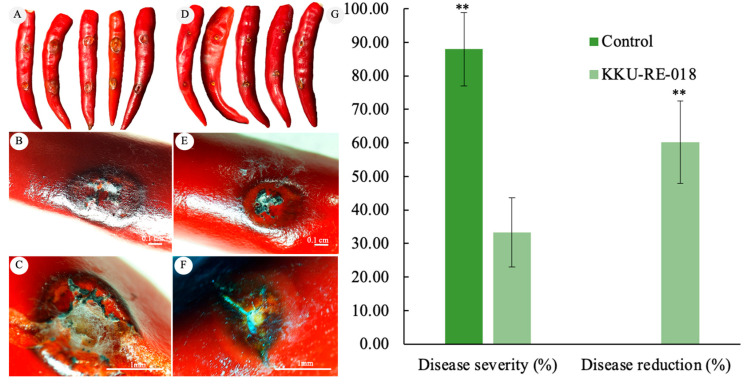
Effect of *Bacillus* sp. KKU-RE-018 on anthracnose disease severity in chili fruits. (**A**–**C**) Disease symptoms in untreated fruits (control); (**D**–**F**) disease symptoms in fruits treated with *Bacillus* sp. KKU-RE-018; (**G**) comparison of percent of disease severity and disease reduction between the treated and control groups. The values are the mean ± SD. Asterisks indicate a significant difference between the control and treatment groups according to the mean values (*p* ≤ 0.01 according to the LSD test).

The in vivo antifungal efficacy of *Bacillus* sp. KKU-RE-018 against the anthracnose pathogen *C. capsici* was evaluated. The disease severity (%) of the *Bacillus* sp. KKU-RE-018-treated plants at 10^8^ CFU·mL^−1^ was reduced more than the non-treated control group at 7 days after inoculation (7 DAI) (Figure 5). The disease severity in the untreated control group reached over 88%, while treatment with *Bacillus* sp. KKU-RE-018 significantly reduced the infection levels to approximately 33.33%. The disease reduction was dramatically reduced to 60.31%, indicating the strong biocontrol potential of strain KKU-RE-018. Visual assessment of the disease symptoms further confirmed the protective effect. The untreated chili fruits exhibited severe anthracnose symptoms, including sunken dark necrotic lesions with sporulation (Figure 5A–C). In contrast, the fruits treated with *Bacillus* sp. KKU-RE-018 showed minimal or no visible symptoms, with the lesions being notably smaller and lacking extensive fungal growth (Figure 5D–F).

### 2.7. Determination of Phenolic Compounds and Salicylic Acid in Chili Fruits

The levels of phenolic compounds and salicylic acid in chili fruits at 7 DAI are shown in Table 3. The results indicate that the chili fruits treated with *Bacillus* sp. KKU-RE-018 exhibited significantly higher levels of both phenolic compounds and salicylic acid than those of the control (*p* < 0.01).

### 2.8. Effects of Bacillus sp. KKU-RE-018 on the Growth of Chili Plants

**Figure 6:**
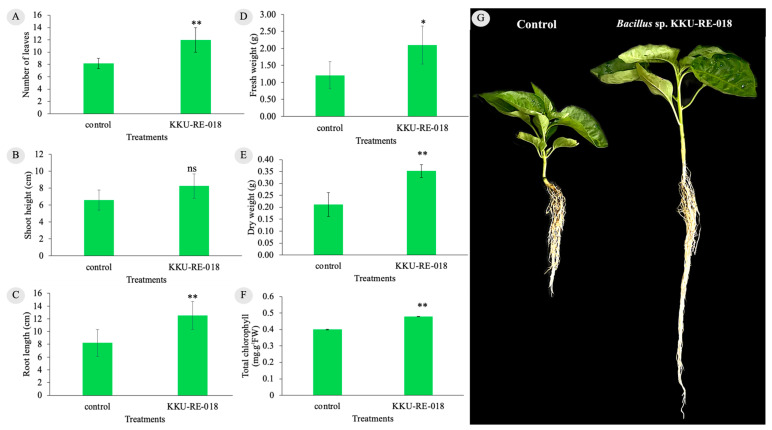
The growth-promoting effect of *Bacillus* sp. KKU-RE-018 on chili plants: (**A**) Number of leaves; (**B**) shoot height; (**C**) root length; (**D**) fresh weight; (**E**) dry weight; (**F**) total chlorophyll; and (**G**) chili plants affected by *Bacillus* sp. KKU-RE-018. The values are the mean ± SD. Asterisks indicate a significant difference between the control and treatment groups according to the mean values. * *p* ≤ 0.05; ** *p* ≤ 0.01, according to the LSD test; ns, non-significant difference.

To investigate the effect of *Bacillus* sp. KKU-RE-018 on chili plants during the seedling stage, several plant growth parameters were assessed at 30 days after inoculation (30 DAI). The parameters, including the number of leaves, shoot height, root length, fresh weight, dry weight, and total chlorophyll, were determined, as shown in Figure 6. The results demonstrated that the plants inoculated with *Bacillus* sp. KKU-RE-018 had a significantly greater increase in plant growth compared to the noninoculated plants (control). The number of leaves of chili seedlings in the control and treatment groups was 8.2 ± 0.84 and 12.0 ± 2.0 cm, respectively (Figure 6A). The height of the shoots was higher in the treated group (8.24 ± 1.44 cm) than in the control (6.58 ± 1.18 cm) (Figure 6B). Similarly, the root length was significantly longer in the treated group (12.50 ± 2.20 cm) compared to the control (8.24 ± 2.11 cm) (Figure 6C). The fresh weight and dry weight showed the same patterns in the treated group at 2.10 ± 0.56 and 0.35 ± 0.03 g, respectively, whereas the control seedlings were 1.21 ± 0.40 and 0.21 ± 0.05 g, respectively (Figure 6D,E). Furthermore, the total chlorophyll was also higher in the treated group (0.48 mg·g^−1^ FW) compared to the control (Figure 6F).

## 3. Discussion

Generally, EPB are widely distributed in nature as an important group of plant-associated microorganisms that colonize the internal tissues of plants without causing harm [21]. They play a dual role in agriculture, serving as both biocontrol agents and PGPs for many plants [22]. In this study, 108 endophytes isolated from medicinal plants were investigated for their inhibition of the growth of *C. capsici*. We found that seven isolates, including KKU-RE-011, KKU-RE-015, KKU-RE-018, KKU-RE-045, KKU-RE-048, KKU-RE-052, and KKU-RE-097, were the most effective against *C. capsici*, in addition to their plant growth-promoting traits. Among them, the isolate KKU-RE-018 demonstrated the highest antagonistic activity, supported by its strong production of hydrolytic enzymes (β-1,3-glucanase and chitinase) and PGP properties, including phosphate solubilization, IAA production, ammonia production, and siderophore production. Through molecular characterization based on the 16S rRNA gene, the isolate KKU-RE-018 was identified as *Bacillus* sp. Members of the genus *Bacillus* are widely reported to contribute to both direct pathogen suppression and plant growth stimulation [23].

The enzyme activity and PGP properties of *Bacillus* sp. KKU-RE-018 occurred through hydrolytic enzymes (chitinase and β-1,3-glucanase), which are considered crucial CWDEs against pathogen fungi. These enzymes are two important proteins that break down chitin and β-1,3-glucan, the major structural components of fungal cell walls [24]. In this study, *Bacillus* sp. KKU-RE-018 was able to produce chitinase and β-1,3-glucanase at considerable levels. The production of these hydrolytic enzymes is consistent with the other *Bacillus* members. For instance, *B. subtilis* LY-1 was reported to produce both chitinase and β-1,3-glucanase, which were associated with significant inhibition of the growth of *Fusarium oxysporum*, *F. proliferatum*, and *F. solani* [25]. Tram et al. [26] demonstrated that *B. velezensis* strain GL7 and strain ML4 exhibited strong β-1,3-glucan activity and were effective in controlling anthracnose disease caused by *Colletotrichum* spp. This suggests that our endophytic bacterium might have great potential against the fungal pathogen through these potent enzymatic mechanisms. In terms of the PGP properties in vitro, *Bacillus* sp. KKU-RE-018 exhibited a remarkable phosphate solubilization capacity. Phosphate-solubilizing endophytes are known to play a role in improving the nutrient availability for host plants and to support plant growth and productivity [27,28]. The solubilization observed in our study aligns with previous findings, supporting the role of endophytic *Bacillus* spp. in phosphorus solubilization [29]. Furthermore, *Bacillus* sp. KKU-RE-018 was able to produce IAA in NB both with supplemented tryptophan and without supplementation, indicating its strong ability to synthesize phytohormones that enhance root elongation and overall plant growth [30,31]. This result agrees with earlier studies, where several *Bacillus* strains, such as *B. siamensis* L17 [32] and *B. subtilis* C-3102 [33], were also found to produce IAA and stimulate plant growth. In addition, KKU-RE-018 produced ammonia and siderophores, both of which contribute to plant growth promotion and pathogen suppression. Ammonia production can provide a supplementary nitrogen source for the development of plant biomass, increased chlorophyll content, and enhanced yield [34], while siderophores enhance iron acquisition and reduce its availability for phytopathogens [35].

In this study, the dual culture assay strongly showed the growth inhibition of the hyphae of *C. capsici*. SEM analysis revealed drastic changes in the external hyphae morphology of *C. capsici*, including shrinkage, distortion, and disruption (Figure 4D,E), in contrast to the smooth and intact hyphae observed in the control (Figure 4C). These results indicate that the suppression of fungal pathogens by *Bacillus* sp. KKU-RE-018 may involve hydrolytic enzymes such as chitinase and β-1,3-glucanase, together with other antifungal metabolites. This is consistent with the findings of Baard et al. [36], who reported that the *B. tequilensis* B2, producing chitinase, was able to inhibit the growth of four plant pathogenic fungi of the genus *Fusarium*, including *F. oxysporum*, *F. culmorum*, *F. proliferatum*, and *F. verticillioides*. Moreover, *B. subtilis* JNF2 was reported to suppress *F. oxysporum* f. sp. through the production of β-1,3-glucanase [37], while *B. mojovensis* B1302 exhibited β-1,3-glucanase against *Rhizoctonia cerealis* [38]. *Bacillus* sp. KKU-RE-018-treated chili plants showed a significant reduction in anthracnose severity, with more than 60% suppression compared with the control. In our greenhouse assay, the disease severity of treated chili fruits was 33.33%. These observations are in line with previous reports of *B. subtilis* strains that were effective in reducing anthracnose in chili. For instance, Le Thanh et al. [39] and Heo et al. [19] demonstrated that *B. subtilis* strains could lower disease severity in both greenhouse and field experiments. Collectively, these findings suggest that *Bacillus* sp. KKU-RE-018 not only inhibits *C. capsici* growth in vitro but also provides effective protection against anthracnose disease under greenhouse conditions and may be applied in the future to suppress anthracnose disease under field conditions.

Interestingly, the enhancement of phenolic compounds and salicylic acid in chili fruit inoculated with *Bacillus* sp. KKU-RE-018 was higher than that of the control. This suggests that *Bacillus* sp. KKU-RE-018 may activate host defense mechanisms. Phenolic compounds are widely recognized as secondary metabolites that contribute to plant resistance by disrupting microbial cell membranes and reinforcing the cell wall at the challenge site [40,41]. Similarly, salicylic acid acts as a crucial signaling molecule in systemic acquired resistance (SAR), acting as a plant defense response triggered by an initial local infection, which results in increased resistance to virulent pathogens in distal uninfected systemic tissues [42]. The significant increase in both phenolic compounds and salicylic acid observed in this study suggests that *Bacillus* sp. KKU-RE-018 may strengthen the defense responses against potential pathogens after an attack by *C. capsici.*

An additional noteworthy finding is the growth-promoting effect of *Bacillus* sp. KKU-RE-018 on chili plants. The result of this study showed that the endophytic bacteria promoted the chili plant parameters significantly more than those of the noninoculated control, including the number of leaves, shoot height, root length, fresh weight, dry weight, and total chlorophyll. This result aligned with the finding of Gowtham et al. [43], who showed that *B. amyloliquefaciens* produced a significant increase in plant height, shoot fresh weight, number of leaves, and shoot dry weight. In addition, treatment with *B. subtilis* L2 markedly enhanced the plant height, number of leaves, leaf length, and leaf width in *Zingiber officinale* Roscoe [44]. Building on these reports, our findings further demonstrate that strain KKU-RE-018 not only enhanced chili growth parameters but also suppressed *C. capsici*, effects that may be associated with CWDE activity and the induction of host defense responses.

## 4. Materials and Methods

### 4.1. Plant Sampling Site

The medicinal plants were collected simultaneously on 14 January 2023, from Nong Phok District, Roi Et (16°9′57″ N, 102°48′12″ E), and Nong Sung District, Mukdahan (16°49′45.6″ N, 100°58′27.6″ E). The plant species sampled included *Chromolaena odorata* (Siam weed), *Phyllanthus urinaria* (chamber bitter)*, Streblus asper* (Siamese rough bush), *Tiliacora triandra* (Yanang), and *Curcuma aromatica* (wild turmeric). Plant samples were randomly collected and then immediately put into plastic bags in an ice box before being transported to the laboratory. The EPB were isolated from the plant samples within 24 h after sampling.

### 4.2. Isolation of EPB

The EPB were isolated from healthy fresh stems, leaves, and roots of medicinal herbs. All sample plants were washed with running tap water to remove the adhered dust and debris; then, the plant organs were cut into small pieces (1 cm) using a sterilized blade, and surface sterilization was conducted according to the modified method of Nxumalo et al. [45]. Briefly, plant segments were immersed in a series of solutions as follows: 75% ethanol for 5 min, (2% *w*/*v*) sodium hypochlorite solution for 4 min, followed by rinsing with sterile distilled water six times and drying under a laminar airflow chamber. To confirm the effectiveness of sterilization, the final rinse water was plated onto nutrient agar (NA). The disinfected sample plants were ground in 6 mL of aqueous saline solution (0.9% NaCl) using a sterilized mortar and pestle under aseptic conditions. The tissue extract was serially diluted in sterilized water. About 100 µL of each diluted (10^−1^–10^−4^) and undiluted sample was pipetted onto NA plates, spread evenly using a sterilized spreader, and incubated at 28 °C for 5 days. The growth colonies were observed daily and selected based on their divergence in morphology, size, and color. They were subcultured twice on NA and stored at 4 °C for further experimentation.

### 4.3. Sources of Pathogenic Fungi

*C*. *capsici* was obtained from a Plant Protection Research and Development office (Bangkok, Thailand). The fungal pathogens were maintained on a potato dextrose agar (PDA) slant for dual culture testing with EPB in future experiments.

### 4.4. Dual Culture Assay

The EPB were tested for the inhibition of pathogen fungi, determined according to the modified method of Zouaoui et al. [46]. Briefly, a culture agar plug (5 mm) of the pure culture pathogen was placed at the edges of a Petri dish containing PDA, which served as a control. The endophyte bacteria were inoculated opposite a culture agar plug (5 mm) of the pathogenic fungus. The cultured plates were incubated at 28 °C for 7 days, and the growth diameter of the pathogen was measured and compared to the control. The percent inhibition of the mycelial growth was calculated by the following formula [47]:

I = (R1 − R2)/R1 × 100,

where I = inhibition of mycelial growth, R1 = growth in control, and R2 = growth in treatment.

### 4.5. Determination of Enzyme Activity Production

#### 4.5.1. Chitinase Activity

Chitinase activity was measured using the method of Khairah et al. [48]. The EPB inoculum was transferred into 20 mL of nutrient broth (NB) and incubated with shaking at 150 rpm, 37 °C for 24 h. After incubation, the culture was centrifuged at 10,000 rpm, at 4 °C for 15 min. The crude extracellular enzyme extract was mixed with 450 μL of 0.3% colloidal chitin and 225 μL of 0.1 M phosphate buffer (pH 7.0) at 37 °C with agitation at 120 rpm for 30 min. The mixture was centrifuged at 10,000 rpm for 15 min, and the supernatant was combined with 750 μL of distilled water and 1500 μL of Schales reagent (K-ferricyanide and 0.5 M Na_2_CO_3_), followed by heating at 100 °C for 10 min. The chitinase activity was determined by measuring the absorbance at 420 nm and using GlcNAc as a standard. One unit of enzyme activity was defined as the amount of enzyme releasing 1 μmol GlcNAc per min.

#### 4.5.2. β-1,3-Glucanase Activity

β-Glucanase activity was determined by quantifying the reducing sugar released from β-glucan as a substrate using the DNS (dinitrosalicylic acid) method [49]. Each bacterial isolate was cultured in 20 mL of NB at 150 rpm, 37 °C for 24 h. The cultures were centrifuged at 10,000 rpm at 4 °C for 15 min, and the supernatant was collected and used as the crude enzyme extract. A volume of 500 µL of crude extract was incubated at 37 °C for 30 min with 500 μL of 3% laminarin (a soluble β-1,3-glucan; Sigma-Aldrich, St. Louis, MO, USA) (dissolved in 50 mM phosphate buffer, pH 7.0). A total of 2 mL of 1% 3,5-dinitrosalicylic acid reagent was added to the sample tube and then boiled for 15 min. The absorbance was measured at a wavelength of 540 nm. The amount of reducing sugar was estimated using glucose as a standard. One unit of β-glucanase activity is defined as the amount of enzyme required to produce 1 µmol of glucose in 1 min of incubation.

### 4.6. Determination of PGP Properties of EPB

#### 4.6.1. Phosphate Solubilization of EPB

The availability of phosphate solubilization for all EPB was determined using Pikovskaya’s agar (PKV) medium (per liter: 0.5 g (NH_4_)_2_SO_4_, 0.1 g MgSO_4_·7H_2_O, 0.02 g NaCl, 0.02 g KCl, 0.003 g FeSO_4_·7H_2_O, 0.003 g MnSO_4_·H_2_O, 10.0 g glucose, 0.5 g yeast extract, and supplemented with 0.5% tricalcium phosphate), following the method of Kuklinsky-Sobral et al. [50]. Then, 2 mL of starter inoculum was added to 20 mL of the PKV medium. The cell cultures were incubated at 150 rpm, 30 °C, for 24 h. The control was made without bacterial inoculation in PKV medium. The cultured broth media were centrifuged at 12,000 rpm for 2 min to remove the bacterial cells. Then, 2 mL of the supernatant was mixed with 5 mL of 2% boric acid, 2 mL of Murphy’s reagent, and 1 mL of 2.5% ascorbic acid, and the final volume at 25 mL was achieved by adding 15 mL of deionized water into the volumetric flask. The solution mixture was maintained at room temperature for 30 min. Thereafter, the absorbance was determined at a wavelength of 820 nm using a spectrophotometer (Hitachi High Tech Science Corporation, Tokyo, Japan). The amount of soluble phosphate was calculated from the standard concentration of potassium dihydrogen phosphate (KH_2_PO_4_).

#### 4.6.2. Indole-3-Acetic Acid (IAA) Production

IAA production was determined from all isolates of EPB using a colorimetric technique performed with Salkowski’s method, according to the method of Cheng et al. [51]. Firstly, 2 mL of EPB inoculum was added to 20 mL of NB supplemented with L-tryptophan (2.0 g·L^−1^) and without L-tryptophan after sterilization. The inoculated broth was incubated at 30 °C on a shaker at 150 rpm for 24 h. A noninoculated medium was set up as a control. After 24 h, the cultures were separated from the supernatant by centrifugation at 12,000 rpm for 2 min. Then, 1 mL of supernatant of each solution was mixed with 2 mL of Salkowski’s reagent (35% perchloric acid and 0.5 M FeCl_3_·6H_2_O; in volume 98 mL: 2 mL (*v*/*v*)) and then incubated in the dark for 30 min. A positive test for indole-related compounds (IRCs) production was indicated by the development of a pink or red color, and the solution mixture was measured using a spectrophotometer at a wavelength of 530 nm. The absorbance values were determined for the quantitative IAA by comparison with the standard curve of the standard IAA.

#### 4.6.3. Ammonia Production

The ammonia production was analyzed using the slightly modified method of Fouda et al. [52]. The freshly grown cultures of endophytic bacterial isolates were cultured in peptone water. The inoculum was incubated at 30 °C with shaking at 150 rpm for 24 h. The control was peptone water without bacterial inoculation. The supernatant was collected via centrifugation at 12,000 rpm for 2 min. Then, 2 mL of Nessler’s reagent was added to each supernatant as a colorimetric reagent for ammonia production. The color change from brown to yellow indicates a positive result for ammonia production. The ammonia quantification was assessed by measuring the absorbance at a wavelength of 530 nm using a spectrophotometer. The ammonia concentration was calculated using a standard curve of ammonium sulfate (NH_4_)_2_SO_4_.

#### 4.6.4. Siderophore Production

Siderophore production was assessed by culturing bacteria on Chrome Azurol S (CAS) agar containing hexadecyltrimethylammonium bromide (HDTMA), prepared by following the modified method of Schwyn and Neilands [53]. The noninoculated plate was used as a control. The plates were incubated at 30 °C for 72 h. The appearance of an orange or yellow halo surrounding the bacterial colonies was considered indicative of siderophore production.

#### 4.6.5. Production of Hydrogen Cyanide (HCN)

Hydrogen cyanide production by the bacterial strains was determined using HCN-sensitive paper [54]. The bacterial isolates were cultured in NA supplemented with 4.4 g·L^−1^ of glycine. Then, Whatman filter paper No. 1 was soaked with a mixture of 0.5% picric acid and 2% sodium carbonate for 1 min before being adhered beneath the Petri dish lids. The plates were sealed with parafilm and incubated at 30 °C for 7 days. After incubation, a change in color of the Whatman filter papers from white to orange or red was indicative of positive HCN production.

### 4.7. Identification of EPB

The EPB isolates were selected for identification, and the analysis of 16S rDNA sequences was carried out for those selected isolates. Genomic DNA was extracted using the freeze–thaw method, as modified by Salo and Novero [55]. DNA purity was assessed using a Nanodrop spectrophotometer (DS-C Cuvette Spectrophotometer, DeNovix Inc., Wilmington, DE, USA), and DNA integrity was verified by agarose gel electrophoresis. For PCR amplification, the universal primers 1427R (5′-CAGTGAACTCCCCCTCCAA-3′) and 27F (5′-AGAGTTTGATCCTGGCTCAG-3′) were used. The reaction conditions included an initial denaturation of 1 min at 94 °C, followed by 30 cycles of denaturation at 94 °C for 30 s, annealing at 55 °C for 30 s, and extension at 72 °C for 30 s. A final extension at 72 °C for 10 min was undertaken at the end of the amplification. PCR products were then purified using the GeneJETTM PCR purification kit (Fermentas, Toronto, ON, Canada). Subsequently, the PCR products were sequenced with the U2Bio company (Seoul, Republic of Korea). The sequence was analyzed by comparing it with sequences in the GenBank database on the National Center for Biotechnology Information (NCBI) website. The closely related sequences were aligned using ClustalW in MEGA 11 software [56]. The neighbor-joining method was used to construct a phylogenetic tree. The bootstrap was used as a statistical support for each node with 1000 replicates.

### 4.8. Scanning Electron Microscopy Analysis of C. capsici Mycelia Under Dual Culture with EPB

Morphological changes in C. *capsici* mycelia were examined by scanning electron microscopy (SEM) after dual culture with EPB. The mycelial discs (5 mm) of the pathogen from the periphery of the inhibition zone in the dual culture plate, from fungal hyphae directly overlaid with the bacterial suspension, as well as in the control plate, were used. The samples were fixed in 1.5% glutaraldehyde and kept for 24 h at 4 °C in a dry Petri dish. Subsequently, the specimens were dehydrated with a graded series of ethanol (10%, 30%, 50%, 70%, 90%, and 100%) and dried in a desiccator. Then, the dried specimens were adhered to double-sided carbon tape and subsequently coated with gold. Finally, they examined under SEM to investigate any morphological changes in the pathogen mycelium compared to the untreated control.

### 4.9. Effect of EPB Against Anthracnose Disease on Chili Fruits

The antifungal activity of EPB on chili fruits was determined by following the method of Liotti et al. [57]. Fresh chili fruits without wounds, scars, and rots on their surface were surface sterilized with 1% sodium hypochlorite solution for 1 min and 70% ethanol for 30 s, followed by rinsing thrice with sterile distilled water. The surface-sterilized chili fruits were wounded to a depth of 1 mm with a sterile needle and subjected to the following treatments: (i) pathogen-inoculated control: chili fruits were inoculated with 5 mm mycelial plugs of pathogen culture; (ii) chili fruits were inoculated with 20 µL of bacterial suspension 1 × 10^8^ CFU·mL^−1^, and after an hour of incubation, 5 mm mycelial plugs of the respective pathogens were placed over them. The chili fruits were placed in separate glass Petri dishes, sealed with parafilm, and incubated at 28 ± 2 °C for 7 days. The experiment was performed with a completely randomized design (CRD) in three replicates of five fruits each.

The level of anthracnose severity on the fruits was visually assessed after 7 days using a 0–4 scale based on the estimated percentage of fruit surface area affected [58], where 0 = 0%, 1 = 1–25%, 2 = 26–50%, 3 = 51–75%, and 4 = 76–100%. The percentage of the disease severity index (DSI) was determined as DSI (%) = (Sum of all numerical ratings/(Quantity of fruits counted × 4)) × 100. The efficacy of the disease reduction index (DRI) of anthracnose severity was DRI (%) = ((DSIcontrol − DSItreatment)/DSIcontrol) × 100. The experiment was conducted in triplicate.

### 4.10. Determination of Phenolic Compounds and Salicylic Acid in Induced Chili Fruits

The phenolic compound and salicylic acid determinations were conducted according to the method described by Le Thanh et al. [39], with some modifications. Briefly, chili fruits (1.0) collected from the previous disease severity assay were subjected to careful grinding with 1 mL of 90% methanol. The layer was separated from the leaves via centrifugation at approximately 12,000 rpm and 4 °C for 5 min and stored immediately at −80 °C in a freezer. The samples were used to perform assays for phenolic compounds and SA.

The content of phenolic compounds in the fruit extracts was determined using the method of Blainski et al. [59]. A volume of 20 µL of the extracts was mixed with 80 µL of 7% sodium carbonate and 100 µL of 10% Folin–Ciocalteau reagent. The mixture was incubated at room temperature for 30 min prior to absorbance measurement at a wavelength of 760 nm. The absorbance values were compared to the standard curve of gallic acid to calculate the quantity of phenolic compounds.

The SA content was determined using the procedure of Warrier et al. [60]. Briefly, 100 µL of fruit extract was mixed with 100 µL of 0.02 M ferric ammonium sulfate. After the mixture was allowed to stand for 5 min, the absorbance was measured at 530 nm. The measured absorbances of the treatment and subsequent reference were used to calculate the amount of SA.

### 4.11. Determination of the PGP Parameter

The EPB isolate was inoculated in NB medium and incubated in a shaking incubator at 150 rpm, 30 °C for 24 h. Cells were harvested by centrifugation at 12,000 rpm for 10 min, washed, and resuspended in sterile distilled water. The cell density was adjusted to 10^8^ CFU·mL^−1^ [61]. Chili seedlings (approximately 2 mm in height), aseptically germinated on Petri plates, were transferred to earthen pots (25 cm × 15 cm) containing a 6 kg mixture of sterilized soil and sand (5:1). After 3 days, each pot was inoculated with 20 mL of the bacterial suspension around the rhizosphere. The control plants received an equal volume of sterile distilled water. Each treatment was conducted in triplicate.

In vivo plant growth promotion was evaluated 30 DAI based on the shoot length, root length, number of leaves, fresh weight, and dry weight were determined. The chlorophyll content was determined using the fresh leaves. Briefly, 1 g of ground leaves was used for chlorophyll extraction using 80% acetone. The mixture was centrifuged at 4000 rpm for 10 min in order to collect the supernatant. Then, Chlorophyll contents were determined by measuring the absorbance at a wavelength of 663 nm (Abs663) and 645 nm (Abs645) using a spectrophotometer (Hitachi High-Tech Science Corporation, Tokyo, Japan). Chlorophyll a, chlorophyll b, and total chlorophyll contents were calculated according to Arnon [62]. For all parameters of each treatment, 6 replications were observed, and the average was calculated.

### 4.12. Statistical Analysis

Analysis of variance (ANOVA) was conducted using the Statistix 10 software. The data were analyzed according to a completely randomized design (CRD) from six plot replications. The least significant difference (LSD) test was applied to test the significant difference among the treatment means at *p* ≤ 0.05.

## 5. Conclusions

The endophytic bacterium *Bacillus* sp. KKU-RE-018, isolated from medicinal plants, showed strong inhibitory effects against *C. capsici* through the production of CWDEs (chitinase and β-1,3-glucanase) and might contribute to enhancing the host plant’s defense system by upregulating the production of phenolic compounds and salicylic acid, which are associated with defense responses. The strain significantly reduced anthracnose disease by over 60% in chili fruit under greenhouse conditions. In addition, this endophytic bacterium possesses PGP traits. These findings highlight that *Bacillus* sp. KKU-RE-018 could be a promising eco-friendly candidate for integrated disease management and sustainable chili production. These results represent a preliminary study under greenhouse conditions. Our research group will further investigate the effectiveness in field trials to confirm its potential for practical application.

## Figures and Tables

**Table 1:** Enzyme activity of EPB isolated from medicinal plants.

EPB Isolates	Enzyme Activity (U·mL^−1^)	
	Chitinase	β-1,3-Glucanase
KKU-RE-011	1.05 ± 0.07 e	1.09 ± 0.01 c
KKU-RE-015	1.59 ± 0.02 c	1.10 ± 0.03 c
KKU-RE-018	1.98 ± 0.05 a **	1.78 ± 0.07 a **
KKU-RE-045	1.47 ± 0.03 d	0.98 ± 0.00 d
KKU-RE-048	1.87 ± 0.06 b	0.92 ± 0.01 e
KKU-RE-052	0.42 ± 0.01 f	1.18 ± 0.01 b
KKU-RE-097	0.22 ± 0.01 g	0.60 ± 0.01 f

Different letters indicate significant differences among values within the same column using the LSD test. **, significantly different (*p* ≤ 0.01 using the LSD test).

**Table 2:** Plant growth-promoting properties of EPB isolated from medicinal plants.

EPB Isolates	Phosphate Solubilization (µg·mL^−1^)	IAA Production (µg·mL^−1^)		NH_3_ Production (µg·mL^−1^)	Siderophore Production (µg·mL^−1^)	HCN Production (µg·mL^−1^)
		+L-tryp	−L-tryp			
KKU-RE-011	714.81 ± 12.90 f	5.21 ± 0.07 d	9.93 ± 1.69 bc	37.51 ± 0.98 b	+	−
KKU-RE-015	1067.13 ± 9.86 a **	4.35 ± 0.35 d	11.81 ± 0.04 bc	33.02 ± 0.51 cd	+	−
KKU-RE-018	918.52 ± 14.85 b	7.36 ± 0.55 c	15.10 ± 1.53 a **	31.59 ± 1.00 d	+	−
KKU-RE-045	766.67 ± 13.39 d	8.52 ± 1.89 c	9.21 ± 2.26 c	42.15 ± 1.23 a **	+	−
KKU-RE-048	776.85 ± 10.52 d	10.69 ± 0.60 b	10.16 ± 2.41 bc	38.70 ± 0.57 b	−	−
KKU-RE-052	861.11 ± 9.10 c	3.82 ± 0.66 d	9.10 ± 1.11 c	33.54 ± 0.52 c	+	−
KKU-RE-097	743.52 ± 14.72 e	19.35 ± 1.10 a **	13.13 ± 1.72 ab	25.33 ± 1.00 e	−	−

Different letters indicate significant differences among values within the same column using the LSD test. **, significantly different (*p* ≤ 0.01 using the LSD test). “+”: Positive result; “−”: negative result.

**Table 3:** Quality of the total phenolic compounds and salicylic acid in chili fruits at 7 DAI.

Treatments	Phenolic (GEA·g^−1^ FW)	Salicylic Acid (µg·g^−1^ FW)
Pathogen-inoculated (control)	16.56	178.11
Pathogen-inoculated + *Bacillus* sp. KKU-RE-018	21.83 **	218.00 **

**, significantly different (*p* ≤ 0.01 using the LSD test).

## Data Availability

No new data were created or analyzed in this study.
